# Modulation of β-Cell Ouabain-Sensitive ^86^Rb^+^ Influx (Na^+^/K^+^ Pump) by D-Glucose, Glibenclamide or Diazoxide

**DOI:** 10.1155/EDR.2000.265

**Published:** 2000

**Authors:** Adrian Elmi, Lars-ÅKe Idahl, Janove Sehlin

**Affiliations:** 1 Department of Integrative Medical Biology Section for Histology and Cell Biology Umeå University Umeå SE-901 87 Sweden

## Abstract

The activity of the β-cell Na^+^/K^+^ pump was studied by using ouabain-sensitive (lmM ouabain) ^86^Rb^+^ influx in β-cell-rich islets of Umeå-*ob/ob* mice as an indicator of the pump function. The present results show that the stimulatory effect of glucose on ouabain-sensitive ^86^Rb^+^ influx reached its approximate maximum at 5mM glucose. Pre-treatment of the islets with 20mM glucose for 60 min strongly reduced the glucose-induced stimulation of the Na^+^/K^+^ pump. Pre-treatment (60 or 180 min) of islets at 0mM glucose, on the other hand, did not affect the magnitude of the glucose-induced stimulation of ^86^Rb^+^ influx dunng the subsequent 5-min incubation. Glibenclamide stimulated the ouabain-sensitive ^86^Rb^+^ uptake in the same manner as glucose. The stimulatory effect, showed its apparent maximum at 0.5μM. Pre-treatment (60 min) of islets with 1μM glibenclamide did not reduce the subsequent stimulation of the ouabain-sensitive ^86^Rb^+^ influx. The stimulatory effect of glibenclamide and D-glucose were not .additive, suggesting that they may have the same mechanism of action. No direct effect of glibenclamide (0.01-1μM) was observed on the Na^+^/K^+^ ATPase activity in homogenates of islets. Diazoxide (0.4mM) inhibited the Na^+^/K^+^ pump. This effect was sustained even after 60 min of pre-treatment of islets with 0.4mM diazoxide. The stimulatory effect of glibenclamide and D-glucose were abolished by diazoxide. It is concluded that nutrient as well as non-nutrient insulin secretagogues activate the Na^+^/K^+^ pump, probably as part of the membrane repolarisation process.


*Int. Jnl. Experimental Diab. Res.*, Vol. 1, pp. 265–274
Reprints available directly from the publisher
Photocopying permitted by license only

© 2001 OPA (Overseas Publishers Association) N.V.
Published by license under
the Harwood Academic Publishers imprint,
part of The Gordon and Breach Publishing Group.
Printed in Malaysia.

### Modulation of $\beta$-Cell Ouabain-Sensitive $^{86}Rb^+$ Influx ($Na^+/K^+$ Pump) by D-Glucose, Glibenclamide or Diazoxide

ADRIAN ELMI, LARS-ÅKE IDAHL and JANOVE SEHLIN*

*Department of Integrative Medical Biology, Section for Histology and Cell Biology, Umeå University, SE-901 87 Umeå, Sweden*

*(Received in final form 28 May 2000)*

**The activity of the $\beta$-cell $Na^+/K^+$ pump was studied by using ouabain-sensitive (1 mM ouabain) $^{86}Rb^+$ influx in $\beta$-cell-rich islets of Umeå-*ob/ob* mice as an indicator of the pump function. The present results show that the stimulatory effect of glucose on ouabain-sensitive $^{86}Rb^+$ influx reached its approximate maximum at 5 mM glucose. Pre-treatment of the islets with 20 mM glucose for 60 min strongly reduced the glucose-induced stimulation of the $Na^+/K^+$ pump. Pre-treatment (60 or 180 min) of islets at 0 mM glucose, on the other hand, did not affect the magnitude of the glucose-induced stimulation of $^{86}Rb^+$ influx during the subsequent 5-min incubation. Glibenclamide stimulated the ouabain-sensitive $^{86}Rb^+$ uptake in the same manner as glucose. The stimulatory effect showed its apparent maximum at 0.5 μM. Pre-treatment (60 min) of islets with 1 μM glibenclamide did not reduce the subsequent stimulation of the ouabain-sensitive $^{86}Rb^+$ influx. The stimulatory effect of glibenclamide and D-glucose were not additive, suggesting that they may have the same mechanism of action. No direct effect of glibenclamide (0.01–1 μM) was observed on the $Na^+/K^+$ ATPase activity in homogenates of islets. Diazoxide (0.4 mM) inhibited the $Na^+/K^+$ pump. This effect was sustained even after 60 min of pre-treatment of islets with 0.4 mM diazoxide. The stimulatory effect of glibenclamide and D-glucose were abolished by diazoxide. It is concluded that nutrient as well as non-nutrient insulin secretagogues activate the $Na^+/K^+$ pump, probably as part of the membrane repolarisation process.**

*Keywords*: Insulin; Sulphonylurea; *ob/ob*-mouse; Ouabain; $Na^+/K^+$ pump; $\beta$-cell

### INTRODUCTION

Stimulation of pancreatic $\beta$-cells by D-glucose or sulphonylurea induces a complex electrical membrane activity, which has a pivot role in activating the secretory machinery.[1] The important role of $K^+$ permeability and activity of $K^+$ channels in the $\beta$-cell plasma membrane is well established (for review see [1]). The ATP-regulated $K^+$ channels dominate the resting conductance of the $\beta$-cell membrane and the glucose-induced closing of these channels leads to the initial depolarisation up to the threshold for generation of an electrical slow wave complex.[2, 3] This model requires the presence of an active $Na^+/K^+$ pump. Early studies of labelled $Rb^+$ transmembrane transport

*Corresponding author. Tel.: +4690-786 51 09, Fax: +4690-786 66 96, e-mail: janove.sehlin@histocel.umu.se showed that β-cells accumulated $^{86}Rb^+$ and extruded $^{22}Na^+$ [4,5] in an ouabain-sensitive manner and indicated that neither glucose[4,6] nor sulphonylureas[7] inhibited the $Na^+/K^+$ pump in pancreatic β-cells. Without finding any direct effect of glucose on the electrogenic $Na^+/K^+$ pump, electrophysiological studies indicated that activation of the $Na^+/K^+$ pump could lead to a marked hyperpolarisation of the β-cell membrane in the presence of 10 mM glucose and that this could be inhibited by ouabain.[8] Direct measurements of islet total content of $Na^+$ by integrating flame photometry have revealed that glucose lowers the islet $Na^+$ content[9] and measurements of cytoplasmic $Na^+$ by the fluorescent probe SBFI (sodium benzofuran isophtalate) indicated a glucose-induced decrease also in the free $Na^+$ activity.[10] These findings indirectly support the idea that the $Na^+/K^+$ pump is activated during glucose stimulation of β-cells. Some previous studies have suggested a direct or indirect inhibitory effect of insulin secretagogues, like glucose and sulphonylureas, on the $Na^+/K^+$ ATPase activity.[11–13]

In the present study we have further characterized the effect of D-glucose, a nutrient secretagogue, and the sulphonylurea glibenclamide, a non-nutrient secretagogue, on islet ouabain-sensitive $^{86}Rb^+$ influx, used as a marker for the $Na^+/K^+$ pump in intact, β-cell-rich islets of Umeå-*ob/ob* mice.

### MATERIALS AND METHODS

### Animals and Isolation of Islets

Adult, non-inbred, 7–8 months old, female *ob/ob* mice from the Umeå colony (Umeå-*ob/ob*), were used throughout the experiments. These animals have hyperplastic islets containing a high proportion of β-cells (>90%),[14] which makes it highly probable that the present data on isolated islets are representative of this cell type.

All mice were fasted overnight but with free access to water, in order to facilitate the isolation of pancreatic islets. The pancreata were digested with collagenase to isolate individual islets as previously described.[15] The function of the isolated islets used in this study was monitored in separate control experiments by measuring the insulin released by islets at different glucose concentrations. Isolated islets used in the study showed an excellent secretory performance (data not shown).

The isolation medium used was a Krebs-Ringer medium (KRH) with the following salt composition (mM): NaCl, 130; KCl, 4.7; $KH_2PO_4$, 1.2; $MgSO_4$, 1.2; and $CaCl_2$, 2.56. Bovine serum albumin (BSA) at 10 mg/ml and 3 mM D-glucose were added. The medium was buffered with 20 mM Hepes and NaOH to a final pH of 7.4.

### Measurements of $^{86}Rb^+$ Influx

Pancreatic islets were isolated as described above. Then, batches of five islets (dry weight 28.9 ± 0.4 μg) were preincubated for 60–180 min at 37°C in KRH medium containing 0, 3 or 20 mM D-glucose and bovine serum albumin (BSA) at 1 mg/ml. After preincubation, islets were incubated for 5 min at 37°C in the same type of basal medium supplemented with 28 μM $^{86}Rb^+$ and 8 μM [6,6′-$^3$H] sucrose as an extracellular marker, essentially as previously described.[4] The test substances were dissolved in the same medium. After incubation the islets were freeze-dried (−40°C, 0.1 Pa), weighed on a quartz-fiber balance and their radioactive contents measured in a liquid scintillation spectrometer.

### Islet Homogenisation

Islets isolated as above were washed four times in a homogenisation medium with the following composition (mM): sucrose, 250; KCl, 50; MOPS, 10; and EGTA, 1. The final pH was set to 7.2 with 2 M Trisma-base. Approximately 100 islets from

*ob/ob* mice were transferred to a polypropylene micro test tube (Milian Instruments S.A., Geneva, Switzerland) and 60 µl homogenisation medium and a glass bead were added. The islets were then homogenised by vibrating the test tube at a frequency of 1 kHz for 30 s followed by a short centrifugation. The test tubes were then immediately frozen at −20°C.

### Assay of $Na^{+}/K^{+}$ ATPase

The method for measuring the activity of $Na^{+}/K^{+}$ ATPase was adapted from Jørgensen and Skou.[16] The concentrations of $Na^{+}$ and $K^{+}$ were chosen to give a maximum rate of ouabain-sensitive ATP hydrolysis (135 mM $Na^{+}$ and 20 mM $K^{+}$). Previous studies on the cationic dependence of the $Na^{+}/K^{+}$ ATPase of mouse pancreatic islets have shown that the enzyme activity at different $Na^{+}/K^{+}$ ratios and the corresponding curve for $Na^{+}$ and $K^{+}$ activation of the $\beta$-cell enzyme (Sandström, Klaerke and Sehlin, unpublished data) are very similar to the data previously described for the $Na^{+}/K^{+}$ ATPase in the kidney.[17,18] The activity of $Na^{+}/K^{+}$ ATPase was measured as the difference in amount of inorganic phosphate released in the presence or absence of ouabain (1 mM). To permeabilize the membranes for measurements of $Na^{+}/K^{+}$ ATPase from pancreatic $\beta$-cells, the homogenate was preincubated with 75 µM DOC for 15 to 30 min at room temperature, according to Jørgensen and Skou.[17]

Incubation was carried out in a histidine buffer with the following final composition (mM): $Na^{+}$, 135; $K^{+}$, 20; $Mg^{2+}$, 2.9; $Cl^{-}$, 149; histidine, 29; and pH 7.4. After the addition of 50 µl ATP (2.9 mM final concentration), pH 7.4 to 450 µl of histidine buffer, the solution was temperature equilibrated for 10 min at 37°C. Then, 25 µl of the preincubated islet homogenate (protein concentration $1.09 \pm 0.10$ µg/µl) was added to the reaction mixture. The reaction was allowed to proceed for 10 min at 37°C. The glibenclamide was dissolved in the histidine buffer and all tests were performed both in the absence and presence of ouabain (1 mM) in parallel with relevant blanks. The reaction was terminated by the addition of 1 ml of ice-cold reagent, containing 150 mM L-ascorbic acid and 3.7 mM ammonium heptamolybdate, on an ice-bath. After 10 min, 1.5 ml of reagent containing 150 mM sodium-*meta*-arsenite, 68 mM sodium citrate and 2% (v/v) acetic acid, was added and the mixture was incubated for 10 min at 37°C. The absorbance of the final solution was then read at 850 nm in a Varian DMS 100 spectrophotometer. Enzyme activity was expressed as mmol inorganic phosphate released (= mmol ATP hydrolysed) per g protein and 10 min. The amount of protein was measured spectrophotometrically as previously described.[19]

### Statistics

Statistical significance was evaluated by using the two-tailed Student's *t*-test for paired data or, when specifically stated, by analysis of variance followed by a Fisher's PLSD test for multiple comparisons. Results are expressed as mean ± S.E.M.

### Chemicals

Amersham Pharmacia Biotech, Uppsala, Sweden provided $^{86}Rb^{+}$ and [6,6′-$^{3}$H] sucrose. Svenska Hoechst AB, Stockholm, Sweden, kindly placed glibenclamide at our disposal. Sodium deoxycholate (DOC), EDTA titriplex II, sodium free, L-histidine, L(+)-ascorbic acid, sodium *meta*-arsenite, sodium citrate and ammonium heptamolybdate were obtained from Merck, Darmstadt, Germany. EGTA, MOPS, ouabain, imidazole, $Na_2HPO_4$, dextrane and collagenase, type I, were all from Sigma Chemical Co., St. Louis, MO, U.S.A. and BSA (fraction V) from Miles, Slough, U.K. Hepes and ATP, disodium salt, were from Boeringer, Mannheim, Germany. All other chemicals were of analytical grade.

### RESULTS

### Effect of D-glucose

To estimate the activity of the $Na^+/K^+$ pump in intact $\beta$-cells, $^{86}Rb^+$ ($K^+$ marker) influx was studied in the absence or presence of 1 mM ouabain. Previous experiments have shown that $^{86}Rb^+$ uptake proceeds linearly for at least 5 min and 1 mM ouabain causes maximum inhibition.[20] Figure 1 shows that the stimulatory effect of glucose on the ouabain-sensitive portion of $^{86}Rb^+$ influx reached its approximate maximum at 5 mM glucose (58%; $n = 14$; $P < 0.001$). The basal rate of ouabain-sensitive $^{86}Rb^+$ influx was thus 63% of the maximum glucose-stimulated rate. The ouabain-resistant portion was significantly and maximally inhibited at 5 mM glucose (37%; $n = 15$; $P < 0.001$). Also the total $^{86}Rb^+$ influx was significantly reduced with an apparent maximum effect at 5 mM glucose (10%; $n = 15$; $P < 0.001$). This is in line with the well established capacity of D-glucose to inhibit $K^+$ channels.

### Pre-treatment with D-glucose

In order to characterise the effects of glucose, the effect of pre-treatment of islets with the sugar on the $^{86}Rb^+$ influx was studied. Sixty min of pre-treatment of islets with 20 mM glucose strongly reduced the glucose-induced stimulation of the ouabain-sensitive portion of $^{86}Rb^+$ influx (Fig. 2, left panel). No statistically significant stimulation above control level was found after this treatment (34% above control; $P > 0.05$; $n = 10$) as compared with 74% above control ($P < 0.001$; $n = 10$) when the islets were preincubated in the

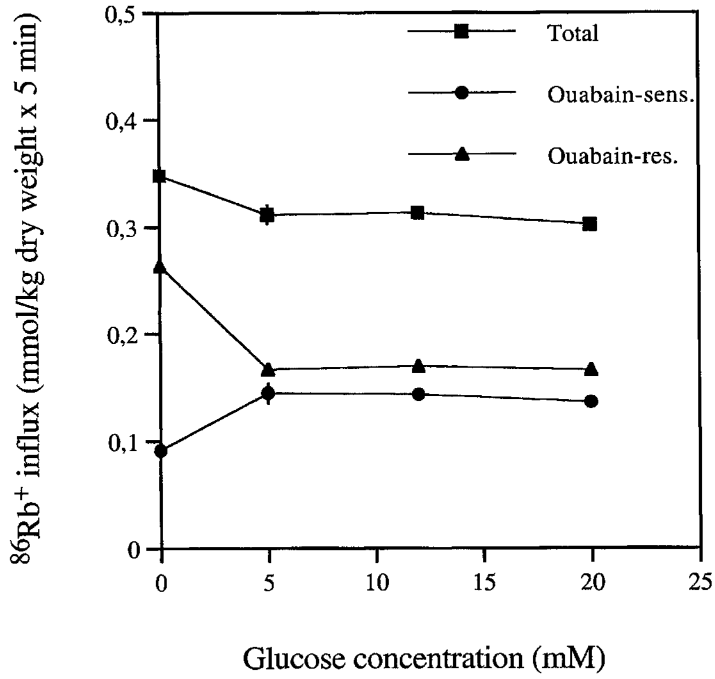

FIGURE 1 *Effect of D-glucose on ouabain-sensitive or ouabain-resistant $^{86}Rb^+$ influx* Pancreatic islets were isolated and preincubated for 60 min in the presence of 3 mM D-glucose as described in the Materials and Methods and then incubated for 5 min at 37°C in the presence of 28 μM $^{86}Rb^+$ and 8 μM [6,6′-$^3$H] sucrose and in the presence of 0, 5, 12 or 20 mM D-glucose and the absence or presence of 1 mM ouabain. Symbols denote mean values ± SEM (bars when larger than symbols) for 16 separate experiments. The data are presented as the total influx of $^{86}Rb^+$ ('Total'), the ouabain-sensitive portion of the influx ('Ouabain-sens.') and the ouabain-resistant portion of the influx ('Ouabain-res.'). $P < 0.001$ for effect of glucose (5–20 mM) within 'Total', 'Ouabain-sens.' and 'Ouabain-res.' respectively (Anova) and $P < 0.001$ for difference between each glucose concentration and respective control (0 mM glucose) within each fraction of $^{86}Rb^+$ uptake (Fisher's PLSD).

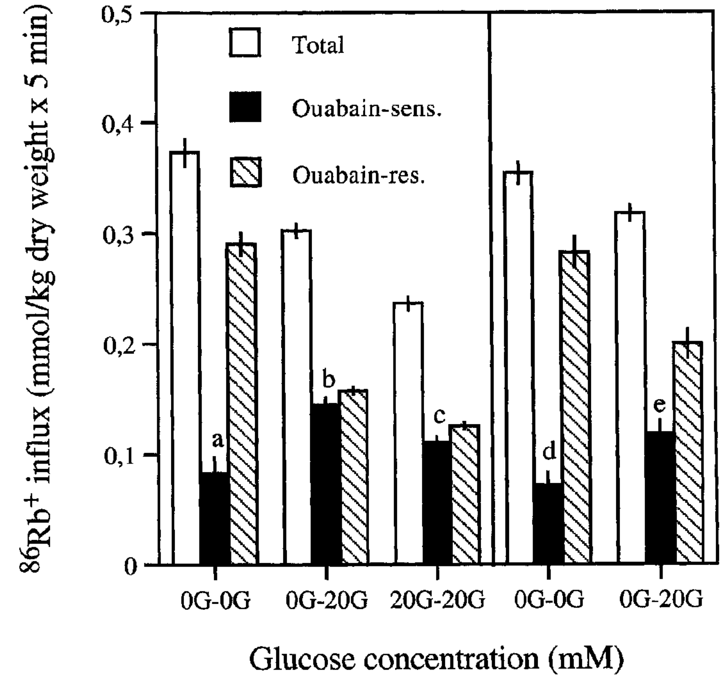

FIGURE 2 *Effect of D-glucose pre-treatment or long-term starvation on ouabain-sensitive or ouabain-resistant $^{86}Rb^+$ influx* Pancreatic islets were isolated and then preincubated for 60 min (left panel) or 180 min (right panel) at 0 or 20 mM glucose. They were then incubated at 37°C in the presence of 28 μM $^{86}Rb^+$ and 8 μM [6,6′-$^3$H] sucrose and in the absence or presence of 20 mM D-glucose and 1 mM ouabain. Bars denote mean values ± SEM for 10 (left panel) or 12 (right panel) separate experiments. The data are presented as the total influx of $^{86}Rb^+$ at 0 or 20 mM D-glucose ('Total'), the ouabain sensitive portion of the influx ('Ouabain-sens.') and the ouabain resistant portion of the influx ('Ouabain-res.'). The terms 0G and 20G refer to the D-glucose concentration during the preincubation (60 or 180 min) and incubation (5 min) periods respectively. For difference between *a* and *b* $P < 0.001$, between *a* and *c* $P > 0.05$ (n.s.), between *b* and *c* $P < 0.01$ and between *d* and *e* $P < 0.05$.

absence of glucose. There is also a significant difference between these two treatments ($P < 0.01$; $n = 10$; for the difference between 34% and 74%) (Fig. 2, left panel). The ouabain-resistant portion was significantly lowered, 57% and 46% respectively ($P < 0.001$; $n = 10$) and the total $^{86}Rb^+$ influx was reduced by 37% and 19% respectively ($P < 0.001$; $n = 10$).

### Effect of Long-term *In Vitro* Fuel Deprivation

To investigate whether long-term fuel deprivation *in vitro* could affect the rate of ouabain-sensitive $^{86}Rb^+$ influx, we used 180 min of preincubation of islets at 0 mM glucose. The results show that this treatment did not affect the level of glucose-induced stimulation of $^{86}Rb^+$ influx during the subsequent 5-min incubation (65%; $P < 0.05$; $n = 12$) as compared with the same treatment for 60 min (Fig. 2). Neither the reduction of the ouabain-resistant portion (−29%; $P < 0.001$; $n = 12$) nor the total influx (−10%; $P < 0.005$; $n = 12$) was different compared to 60 min of treatment.

It should be noted that the changes in different portions of the $^{86}Rb^+$ influx were similar, irrespective of the preincubation time (60 or 180 min) or glucose concentration during the preincubation time (0 or 3 mM).

### Effect of Glibenclamide

The effect of the hypoglycaemic sulphonylurea, glibenclamide, on islet-ouabain-sensitive $^{86}Rb^+$ influx was studied by analysing the effect of a range of glibenclamide concentrations (0.1 to 10 μM) in the absence or presence of 1 mM ouabain. Glibenclamide, like glucose, stimulated the $Na^+/K^+$ pump (28%; $P < 0.05$; $n = 6$). This stimulatory effect was evident already at 0.5 μM and did not further increased in magnitude with increasing concentration of glibenclamide (Tab. I). Glibenclamide caused a dose-dependent inhibition of the ouabain-resistant $^{86}Rb^+$ influx (Tab. I). Pre-treatment (60 min) of islets with 1 μM glibenclamide did not reduce the subsequent stimulation of the ouabain-sensitive or inhibition of ouabain-resistant $^{86}Rb^+$ influx (Tab. II), as compared with exposure to glibenclamide for only 5 min (Tab. III). The stimulatory effect of glibenclamide, on the ouabain-sensitive $^{86}Rb^+$ influx, was not additive to that caused by D-glucose (Tab. III).

TABLE I Effect of glibenclamide on $^{86}Rb^+$ influx

| Glibenclamide conc. (μM) | $^{86}Rb^+$ influx (mmol/kg dry weight) Primary data | Difference from control |
|---|---|---|
| Ouabain-sensitive | | |
| Control (0) | 0.1119 ± 0.017 (6) | – |
| 0.1 | 0.1324 ± 0.009 (6) | 0.0204 ± 0.010 n.s. |
| 0.5 | 0.1422 ± 0.013 (6) | 0.0303 ± 0.012 $P < 0.05$ |
| 1.0 | 0.1402 ± 0.012 (6) | 0.0283 ± 0.011 $P < 0.05$ |
| 10.0 | 0.1442 ± 0.010 (6) | 0.0322 ± 0.011 $P < 0.05$ |
| Ouabain-resistant | | |
| Control (0) | 0.2810 ± 0.016 (6) | – |
| 0.1 | 0.1947 ± 0.008 (6) | −0.0862 ± 0.012 $P < 0.001$ |
| 0.5 | 0.1408 ± 0.008 (6) | −0.1402 ± 0.009 $P < 0.001$ |
| 1.0 | 0.1248 ± 0.004 (6) | −0.1562 ± 0.014 $P < 0.001$ |
| 10.0 | 0.0965 ± 0.001 (6) | −0.1845 ± 0.015 $P < 0.001$ |

Islets were prepared as described in the materials and methods and then preincubated for 60 min at 37°C in KRH medium containing 3 mM glucose but no glibenclamide. They were then incubated for 5 min at 37°C in the absence of glucose but presence of different concentrations of glibenclamide. Data is expressed as mean values for primary data and difference from control ± SEM for the number of experiments indicated in parentheses. n.s. denotes $P > 0.05$ for difference from control.

TABLE II Effect of pre-treatment with glibenclamide or diazoxide on $^{86}Rb^+$ influx

| Test substance | $^{86}Rb^+$ influx (mmol/kg dry weight) Primary data | Difference from control |
|---|---|---|
| Ouabain-sensitive | | |
| Control (0) | 0.1016 ± 0.014 (8) | – |
| Glibenclamide (1.0 μM) | 0.1445 ± 0.006 (8) | 0.0429 ± 0.014 $P < 0.02$ |
| Diazoxide (0.4 mM) | 0.0697 ± 0.007 (8) | −0.0319 ± 0.012 $P < 0.05$ |
| Ouabain-resistant | | |
| Control (0) | 0.2508 ± 0.013 (8) | – |
| Glibenclamide (1.0 μM) | 0.0680 ± 0.003 (8) | −0.1827 ± 0.014 $P < 0.001$ |
| Diazoxide (0.4 mM) | 0.3646 ± 0.005 (8) | 0.1139 ± 0.011 $P < 0.001$ |

Islets were prepared as described in the materials and methods and then preincubated for 60 min at 37°C in KRH medium containing 3 mM glucose alone or 3 mM glucose as well as 1 μM glibenclamide or 0.4 mM diazoxide. The islets were then incubated for 5 min at 37°C in the absence of glucose but presence of 1 μM glibenclamide or 0.4 mM diazoxide. Data is expressed as mean values for primary data and difference from control ± SEM for the number of experiments indicated in parentheses.

TABLE III Effect of glibenclamide, diazoxide or D-glucose on $^{86}Rb^+$ influx

| Test substance | $^{86}Rb^+$ influx (mmol/kg dry weight) Primary data | Difference from control |
|---|---|---|
| Ouabain-sensitive | | |
| Control (0) | 0.1131 ± 0.006 (37) | – |
| Glibenclamide (1.0 μM) | 0.1314 ± 0.004 (29) | 0.0181 ± 0.007 $P < 0.02$ |
| Diazoxide (0.4 mM) | 0.0823 ± 0.008 (23) | −0.0314 ± 0.010 $P < 0.005$ |
| D-glucose (20 mM) | 0.1588 ± 0.005 (16) | 0.0464 ± 0.009 $P < 0.001$ |
| Glibenclamide + Diazoxide | 0.1107 ± 0.007 (15) | −0.0037 ± 0.012 n.s. |
| D-glucose + Diazoxide | 0.1344 ± 0.017 (8) | 0.0221 ± 0.019 n.s. |
| D-glucose + Glibenclamide | 0.1651 ± 0.006 (8)# | 0.0527 ± 0.006 $P < 0.001$ |
| Ouabain-resistant | | |
| Control (0) | 0.2616 ± 0.006 (37) | – |
| Glibenclamide (1.0 μM) | 0.1207 ± 0.002 (29) | −0.1410 ± 0.006 $P < 0.001$ |
| Diazoxide (0.4 mM) | 0.4100 ± 0.008 (23) | 0.1504 ± 0.009 $P < 0.001$ |
| D-glucose (20 mM) | 0.1512 ± 0.004 (16) | −0.1058 ± 0.010 $P < 0.001$ |
| Glibenclamide + Diazoxide | 0.1917 ± 0.006 (15)* | −0.0670 ± 0.008 $P < 0.001$ |
| D-glucose + Diazoxide | 0.2000 ± 0.006 (8)* | −0.0612 ± 0.014 $P < 0.005$ |
| D-glucose + Glibenclamide | 0.1252 ± 0.002 (8) | −0.1276 ± 0.014 $P < 0.001$ |

Islets were prepared as described in the materials and methods and then preincubated for 60 min at 37°C in KRH medium containing 3 mM glucose alone. They were then incubated for 5 min at 37°C in the absence of glucose but presence of 1 μM glibenclamide, 0.4 mM diazoxide, 20 mM D-glucose or combinations of those. Data is expressed as mean values for primary data and difference from control ± SEM for the number of experiments indicated in parentheses. n.s. denotes $P > 0.05$ for difference from control.

* $P < 0.001$ for difference between glibenclamide + diazoxide and glibenclamide alone and D-glucose + Diazoxide and D-glucose alone.

\# n.s. for difference between D-glucose alone and D-glucose + Glibenclamide.

TABLE IV Effect of glibenclamide on $Na^+/K^+$ ATPase activity in islets of *ob/ob* mice

| Glibenclamide conc. (μM) | $Na^+/K^+$ ATPase activity (mmol ATP/g protein and 10 min) Primary data | Difference from control | Students *t*-test paired | Students *t*-test unpaired |
|---|---|---|---|---|
| 0 | 0.287 ± 0.012 (16) | – | | |
| 0.01 | 0.251 ± 0.019 (7) | 0.010 ± 0.014 | n.s. | n.s. |
| 0.1 | 0.286 ± 0.006 (7) | −0.025 ± 0.020 | n.s. | n.s. |
| 1.0 | 0.295 ± 0.012 (16) | −0.009 ± 0.012 | n.s. | n.s. |
| 50 | 0.271 ± 0.011 (9) | 0.035 ± 0.013 | $P < 0.05$ | n.s. |

Homogenates of *ob/ob*-mouse islets were prepared and preincubated as described in the materials and methods and then incubated for 10 min at 37°C in the presence of different concentrations of glibenclamide. Data is expressed as mean values for primary data and difference from control ± SEM for the number of experiments indicated in parentheses. n.s. denotes $P > 0.05$ for difference from control.

Earlier results showed that glibenclamide inhibited the activity of the $Na^+/K^+$ ATPase in HIT-cells by as much as 40% at a drug concentration of 50 nM.[12] We studied the effect of glibenclamide on ouabain-sensitive ATP hydrolysis in islet homogenates by using concentrations of the drug ranging from 0 to 50 μM in order to characterise the proposed effect on $Na^+/K^+$ ATPase of pancreatic β-cells. Table IV shows that no significant effect of glibenclamide on the ATPase activity was observed at drug concentrations up to 1 μM, whereas 50 μM caused a small reduction of probable statistical significance (Tab. IV).

### Effect of Diazoxide

The effect of diazoxide on the rate of $^{86}Rb^+$ influx was studied. Diazoxide at 0.4 mM inhibited the ouabain-sensitive $^{86}Rb^+$ influx by 38% ($P < 0.005$; $n = 15$), while the ouabain-resistant portion was stimulated (62%; $P < 0.001$; $n = 15$) and the total influx was increased (31%; $P < 0.001$; $n = 15$) (Tab. III). The inhibitory effect of diazoxide (0.4 mM) was sustained even after 60 min of pre-treatment of islets with the drug (Tab. II). Diazoxide (0.4 mM), administrated simultaneously with glibenclamide (1.0 µM) totally abolished the stimulatory effect exerted by glibenclamide on the ouabain-sensitive $^{86}Rb^+$ influx (Tab. III). The inhibitory effect of glibenclamide on the ouabain-resistant portion was, however, only partially restored by diazoxide (Tab. III). The stimulatory effect of D-glucose on the ouabain-sensitive $^{86}Rb^+$ influx was reversed by diazoxide (Tab. III).

### DISCUSSION

The present results show that D-glucose, already at low concentrations (5 mM), causes maximum stimulation of the $Na^+/K^+$ pump, as indicated by the ouabain-sensitive $^{86}Rb^+$ influx. This finding is in accordance with previous results indicating that D-glucose lowers the islet $Na^+$ content in an ouabain-sensitive manner [9, 21] and that this effect also appears to reach its maximum at 5 mM glucose.[21] Also, the present results are in accord with the previous finding of glucose-induced increase in the rate of fractional outflow of $^{22}Na^+$ in islets,[22] although this effect was not abolished by ouabain.[22]

The $Na^+/K^+$ pump participates in the generation of the resting membrane potential in pancreatic $\beta$-cells.[8] It has been suggested that as much as 75–80% of the basal energy production in the endocrine pancreas is consumed by the $Na^+/K^+$ pump.[23] Our results show that the $Na^+/K^+$ pump is active even in the absence of glucose when the $\beta$-cells are resting. This basal activity amounts to more than 60% of the maximum $Na^+/K^+$ pump activity seen in the presence of glucose. One explanation to consider for the glucose-induced stimulation of the $Na^+/K^+$ pump in the present results could be that incubation in the absence of glucose causes a relative ATP deficiency and that the observed glucose effect simply represents a difference from starvation. If this was the case, one would expect that prolonging the treatment period in the absence of glucose from 5 min to 60 or 180 min should further aggravate this condition. However, when the islets were exposed to prolonged *in vitro* starvation (0 mM glucose) we found no further change in the $Na^+/K^+$ pump activity. The ATP supply in the $\beta$-cells seemed to be enough to support the basal activity of the $Na^+/K^+$ pump and to allow a seemingly normal glucose-stimulation of the pump after 180 min of *in vitro* starvation. It thus seems unlikely that the glucose effect is mainly due to increased metabolism-induced ATP production. It seems more likely that the depolarisation of the $\beta$-cell by D-glucose stimulates the $Na^+/K^+$ pump as part of the repolarisation of the cell. This idea is supported by the observations that the stimulatory glucose effect on the pump was reversed by diazoxide and that the effects of glucose and glibenclamide were not additive.

The present observation that pre-treatment of islets with a high glucose concentration (20 mM) decreases the subsequent glucose-induced stimulation of the $Na^+/K^+$ pump is of interest, suggesting that this glucose effect is relatively transient. This may indicate that the $Na^+/K^+$ pump is activated by glucose mainly during the acute, first phase of glucose stimulation. The apparent discrepancy between the present results and earlier reports, indicating that the glucose-induced lowering of the $Na^+$ content is not attributable to the activation of the $Na^+/K^+$ pump,[9] could, at least in part, be explained by the transient appearance of the glucose effect, since the islets were pre-treated with the sugar for 60 min.[9] It could be difficult to identify the remaining part of a transient glucose effect on the pump. Of course, glucose is likely to affect the $\beta$-cell $Na^+$ content in several ways.

Sulphonylureas are thought to act mainly by stimulating the pancreatic $\beta$-cells secretion of insulin.[24] It has been shown that these drugs affect several parameters in the islets, such as to reduce $^{86}Rb^+$ permeability,[25, 26] increase $^{36}Cl^-$ permeability,[27] increase $^{45}Ca^{2+}$ uptake,[26, 28, 29] and depolarise the $\beta$-cell membrane. [30] It has also been proposed that the sulphonylurea glibenclamide, directly inhibits the $Na^+/K^+$ ATPase in $\beta$-cells.[12] We were not able to detect any direct inhibitory effect of glibenclamide on the $Na^+/K^+$ ATPase at 0.01 – 1.0 μM of the drug. In this concentration range glibenclamide stimulates the $\beta$-cell secreting machinery.[31] The slight inhibitory effect of glibenclamide detected at the highest drug concentration is of questionable statistical significance and could be due to a direct interaction with the lipid membrane. [32]

The present finding of a stimulatory effect of glibenclamide on the $Na^+/K^+$ pump is in line with the idea of a role for the $Na^+/K^+$ pump in electrical repolarisation. Sulphonylureas cause insulin secretion by closing the ATP-sensitive $K^+$-channels and thereby depolarising the $\beta$-cells. [33, 34] The observation of similar effects of D-glucose and glibenclamide on the $Na^+/K^+$ pump supports the idea that agents, which are causing electrical depolarisation of the $\beta$-cells, also indirectly stimulate the $Na^+/K^+$ pump as part of the repolarisation process. This idea is supported by the fact that diazoxide, an opener of ATP-sensitive $K^+$-channels [34, 35] and inhibitor of insulin secretion by hyperpolarising the $\beta$-cells, [36] inhibited the basal $Na^+/K^+$ pump activity. When glibenclamide and diazoxide were added in combination, the pump activity returned to control level. This could be explained by the opposite effects of these drugs on $K^+_{ATP}$ channel activity and thereby on the membrane potential. The idea is also supported by previous studies indicating that diazoxide reversed the tolbutamide-induced increase in $[Ca^{2+}]_i$, while it did not affect the rise in $[Ca^{2+}]_i$ caused by high $K^+$.[37] Previous studies have shown that in contrast to the effect of D-glucose, tolbutamide did not lower the total islet $Na^+$ content[9] and indeed slightly increased the intracellular $Na^+$ activity,[10] which may partly be due to stimulated entry of $Na^+$.[38]

The present results show that both nutrient and non-nutrient insulin secretagogues, which act by depolarising the $\beta$-cell membrane, also stimulate the ouabain-sensitive $^{86}Rb^+$ influx. The stimulation is abolished by diazoxide. We are unable to show any direct effects of D-glucose[39] or glibenclamide (this study) on the $Na^+/K^+$ ATPase activity in islet homogenates. We therefore suggest that the activation of the $Na^+/K^+$ pump by those secretagogues occurs secondary to membrane depolarisation and that it may contribute to membrane repolarisation.

### *Acknowledgements*

This work was supported in part by the Swedish Medical Research Council (12X-4756), the Swedish Diabetes Association and the Elsa and Folke Sahlberg Fund.

### *References*

[1] Ashcroft, F. M. and Rorsman, P. (1989). Electrophysiology of the pancreatic $\beta$-cell. *Prog. Biophys. Mol. Biol.*, **54**, 87–143.

[2] Ashcroft, F. M., Harrison, D. E. and Ashcroft, S. J. (1984). Glucose induces closure of single potassium channels in isolated rat pancreatic $\beta$-cells. *Nature*, **312**, 446–448.

[3] Rorsman, P. and Trube, G. (1985). Glucose dependent $K^+$-channels in pancreatic $\beta$-cells are regulated by intracellular ATP. *Pflügers Arch.*, **405**, 305–309.

[4] Sehlin, J. and Täljedal, I.-B. (1974). Transport of rubidium and sodium in pancreatic islets. *J. Physiol. (Lond.)*, **242**, 505–515.

[5] Sehlin, J. and Täljedal, I.-B. (1974). Sodium uptake by microdissected pancreatic islets: effects of ouabain and chloromercuribenzene-*p*-sulphonic acid. *FEBS Lett.*, **39**, 209–213.

[6] Lebrun, P., Malaisse, W. J. and Herchuelz, A. (1983). $Na^+-K^+$ pump activity and the glucose-stimulated $Ca^{2+}$-sensitive $K^+$ permeability in the pancreatic $\beta$-cell. *J. Membr. Biol.*, **74**, 67–73.
[7] Lebrun, P., Malaisse, W. J. and Herchuelz, A. (1985). Do hypoglycemic sulfonylureas inhibit $Na^+$, $K^+$-ATPase activity in pancreatic islets? *Am. J. Physiol.*, **248**, E491–E499.
[8] Henquin, J. C. and Meissner, H. P. (1982). The electrogenic sodium–potassium pump of mouse pancreatic $\beta$-cells. *J. Physiol. (Lond.)*, **332**, 529–552.
[9] Wesslén, N. and Hellman, B. (1988). Characterization of the glucose-induced lowering of sodium in mouse pancreatic $\beta$-cells. *Acta Physiol. Scand.*, **133**, 11–17.
[10] Ali, L., Grapengiesser, E., Gylfe, E., Hellman, B. and Lund, P.-E. (1989). Free and bound sodium in pancreatic $\beta$-cells exposed to glucose and tolbutamide. *Biochem. Biophys. Res. Commun.*, **164**, 212–218.
[11] Levin, S. R., Kasson, B. G. and Driessen, J. F. (1978). Adenosine triphosphatases of rat pancreatic islets: comparison with those of rat kidney. *J. Clin. Invest.*, **62**, 692–701.
[12] Ribalet, B., Mirell, C. J., Johnson, D. G. and Levin, S. R. (1996). Sulfonylurea binding to a low-affinity site inhibits the Na/K-ATPase and the $K_{ATP}$ channel in insulin-secreting cells. *J. Gen. Physiol.*, **107**, 231–241.
[13] Owada, S., Larsson, O., Arkhammar, P., Katz, A. I., Chibalin, A. V., Berggren, P.-O. and Bertorello, A. M. (1999). Glucose decreases $Na^+$, $K^+$-ATPase activity in pancreatic $\beta$-cells. An effect mediated *via* $Ca^{2+}$-independent phospholipase $A_2$ and protein kinase C-dependent phosphorylation of the $\alpha$-subunit. *J. Biol. Chem.*, **274**, 2000–2008.
[14] Hellman, B. (1965). Studies in obese-hyperglycemic mice. *Ann. N. Y. Acad. Sci.*, **131**, 541–558.
[15] Lernmark, Å. (1974). The preparation of, and studies on, free cell suspensions from mouse pancreatic islets. *Diabetologia*, **10**, 431–438.
[16] Jørgensen, P. L. and Skou, J. C. (1969). Preparation of highly active $(Na^+ + K^+)$-ATPase from the outer medulla of rabbit kidney. *Biochem. Biophys. Res. Commun.*, **37**, 39–46.
[17] Jørgensen, P. L. and Skou, J. C. (1971). Purification and characterization of $(Na^+ + K^+)$-ATPase. I. The influence of detergents on the activity of $(Na^+ + K^+)$-ATPase in preparations from the outer medulla of rabbit kidney. *Biochem. Biophys. Acta*, **233**, 366–380.
[18] Jørgensen, P. L. (1980). Sodium and potassium ion pump in kidney tubules. *Physiol. Rev.*, **60**, 864–917.
[19] Whitaker, J. R. and Granum, P. E. (1980). An absolute method for protein determination based on difference in absorbance at 235 and 280 nm. *Anal. Biochem.*, **109**, 156–159.
[20] Lindström, P., Norlund, L. and Sehlin, J. (1986). Glucose reduces both $Rb^+$ influx and efflux in pancreatic islet cells. *FEBS Lett.*, **200**, 67–70.
[21] Wesslén, N., Bergsten, P. and Hellman, B. (1986). Glucose-induced reduction of the sodium content in $\beta$-cell-rich pancreatic islets. *Biosci. Rep.*, **6**, 967–972.
[22] Kawazu, S., Boschero, A. C., Delcroix, C. and Malaisse, W. J. (1978). The stimulus-secretion coupling of glucose-induced insulin release. XXVIII. Effect of glucose on $Na^+$ fluxes in isolated islets. *Pflügers Arch.*, **375**, 197–206.
[23] Malaisse, W. J., Boschero, A. C., Kawazu, S. and Hutton, J. C. (1978). The stimulus secretion coupling of glucose-induced insulin release. XXVII. Effect of glucose on $K^+$ fluxes in isolated islets. *Pflügers Arch.*, **373**, 237–242.
[24] Hellman, B. and Täljedal, I.-B. (1975). Effects of sulphonylurea derivatives on pancreatic $\beta$-cells. In: *Handbook of experimental pharmacology*, Edited by Hasselblatt, A. and Bruchhausen, F. V., pp. 175–194, New series, vol. XXXII/2, Berlin, Heidelberg, New York, Springer-Verlag.
[25] Boschero, A. C. and Malaisse, W. J. (1979). Stimulus-secretion coupling of glucose-induced insulin release. XXIX. Regulation of $^{86}Rb^+$ efflux from perfused islets. *Am. J. Physiol.*, **236**, E139–E146.
[26] Henquin, J. C. (1980). Tolbutamide stimulation and inhibition of insulin release: studies of the underlying ionic mechanisms in isolated rat islets. *Diabetologia*, **18**, 151–160.
[27] Sehlin, J. (1981). Are $Cl^-$ mechanisms in mouse pancreatic islets involved in insulin release? *Ups. J. Med. Sci.*, **86**, 177–182.
[28] Malaisse, W. J., Mahy, M., Brisson, G. R. and Malaisse-Lagae, F. (1972). The stimulus-secretion coupling of glucose-induced insulin release. VIII. Combined effects of glucose and sulfonylureas. *Eur. J. Clin. Invest.*, **2**, 85–90.
[29] Hellman, B., Lenzen, S., Sehlin, J. and Täljedal, I.-B. (1977). Effects of various modifiers of insulin release on the lanthanum- nondisplaceable $^{45}Ca^{2+}$ uptake by isolated pancreatic islets. *Diabetologia*, **13**, 49–53.
[30] Meissner, H. P. and Atwater, I. J. (1976). The kinetics of electrical activity of beta cells in response to a "square wave" stimulation with glucose or glibenclamide. *Horm. Metab. Res.*, **8**, 11–16.
[31] Norlund, L. and Sehlin, J. (1984). The acyl-amino-alkyl benzoic acid residue and the sulfonylurea containing residue of glibenclamide affect different aspects of $\beta$-cell function. *Acta Physiol. Scand.*, **120**, 283–286.
[32] Ariens, E. J. (1969). [Oral antidiabetics. Dose, plasma concentration and effect]. *Acta Diabetol. Lat.*, **6** (Suppl. 1:143-76), 143–176.
[33] Sturgess, N. C., Ashford, M. L., Cook, D. L. and Hales, C. N. (1985). The sulphonylurea receptor may be an ATP-sensitive potassium channel. *Lancet*, **2**, 474–475.
[34] Trube, G., Rorsman, P. and Ohno-Shosaku, T. (1986). Opposite effects of tolbutamide and diazoxide on the ATP-dependent $K^+$ channel in mouse pancreatic beta-cells. *Pflügers Arch.*, **407**, 493–499.
[35] Dunne, M. J., Illot, M. C. and Peterson, O. H. (1987). Interaction of diazoxide, tolbutamide and $ATP^{4-}$ on nucleotide-dependent $K^+$ channels in an insulin-secreting cell line. *J. Membr. Biol.*, **99**, 215–224.
[36] Henquin, J. C. and Meissner, H. P. (1982). Opposite effects of tolbutamide and diazoxide on $^{86}Rb^+$ fluxes and membrane potential in pancreatic $\beta$ cells. *Biochem. Pharmacol.*, **31**, 1407–1415.
[37] Grapengiesser, E., Gylfe, E. and Hellman, B. (1990). Sulfonylurea mimics the effect of glucose in inducing large amplitude oscillations of cytoplasmic $Ca^{2+}$ in pancreatic $\beta$-cells. *Mol. Pharmacol.*, **37**, 461–467.
[38] Saha, S. and Grapengiesser, E. (1995). Glucose promotes turnover of $Na^+$ in pancreatic $\beta$-cells. *Biochim. Biophys. Acta*, **1265**, 209–212.
[39] Elmi, A., Idahl, L.-Å., Sandström, P.-E. and Sehlin, J. (2000). D-glucose stimulates the $Na^+/K^+$ pump in mo- use pancreatic islet cells. *Int. J. Exp. Diab. Res.*, In press.

